# Identification of candidate nucleomodulins in ESKAPE bacteria – *in silico* prediction of bacterial proteins harboring canonical nuclear localization sequences

**DOI:** 10.3389/fcimb.2026.1840752

**Published:** 2026-06-15

**Authors:** Laura Riedl, Filip Vilotic, Luzia Burger, Annika Walter, Juergen Fritsch

**Affiliations:** 1 Department of Infection Prevention and Infectious Diseases, University Hospital of Regensburg, Regensburg, Germany; 2 University of Regensburg, Regensburg, Germany

**Keywords:** *A. baumannii*, *E. faecalis*, *E. faecium*, *H. pylori*, *K. pneumoniae*, nuclear import, nucleomodulin, *S. aureus*

## Abstract

An increasing number of bacteria are known to adopt intracellular lifestyles. The intracellular niche protects them from the host immune system or antibiotic treatment in human or animal hosts, while providing access to essential nutritional resources. Bacteria can actively modulate the molecular and structural architecture of the intracellular environment to make it hospitable, e.g. by secreting proteins into the cells. For some of the secreted proteins, translocation into the host cell nucleus and direct targeting of nuclear processes have been described; hence, they are termed nucleomodulins (NMs). In this study, we performed an *in silico* approach to predict putative NMs in *S. aureus*, *E. faecium*, *E. faecalis*, *K. pneumoniae*, *A. baumannii* and *H. pylori*. The results reveal the presence of proteins encoding classical nuclear localization sequences (cNLS), conferring the ability to interact with nuclear importins. This set of candidate NMs will require experimental validation to reveal pathogen-host interactions via direct nuclear targeting.

## Background

Nucleomodulins are a recently defined family of bacterial effector proteins that translocate into the host cell nucleus, where they directly modulate chromatin structure, transcriptional regulation, and epigenetic states to support pathogen survival. The term was initially coined to describe bacterial effectors that mimic eukaryotic nuclear proteins (Bierne and Cossart, 2012). The first NMs were identified in phytopathogens, such as *Agrobacterium tumefaciens* and *Xanthomonas* species, where VirD2 and TAL effectors function as transcriptional regulators or DNA-integrating factors (Ream, 2009).

For mammalian pathogens, NMs are now known across diverse taxa: *Listeria monocytogenes* (LntA, OrfX) (Lebreton et al., 2014; Prokop et al., 2017), *Legionella pneumophila* (RomA, LegAS4, SnpL, AnkH) (Chung et al., 2021; Monteiro et al., 2022; Rolando et al., 2013; Schuelein et al., 2018), *Anaplasma phagocytophilum* (AnkA) (Sinclair et al., 2015), *Ehrlichia chaffeensis* (TRP32, TRP47, TRP120) (Wakeel et al., 2011), *Mycobacterium tuberculosis* (Rv1988, Rv3423, Rv2966c) (Jose et al., 2016; Sharma et al., 2015; Yaseen et al., 2015), *Chlamydia trachomatis* (NUE) (Pennini et al., 2010), *Shigella flexneri* (OspF, Osp1C, IpaH9.8) (Ashida et al., 2010; Haraga and Miller, 2003; Zurawski et al., 2006), *Salmonella enterica* (SspH1, PipA, GtgA, GogA) (Haraga and Miller, 2003; Takemura et al., 2021), and *Helicobacter pylori* (HP0425, HP0059, UreA) (Kim et al., 2016; Kwon et al., 2016; Lee et al., 2015). Additional NMs have been identified in other clinically relevant human pathogens, including *Acinetobacter baumannii* (OmpA, Tnp) (Choi et al., 2008; Moon et al., 2012), *Klebsiella pneumoniae* (HsdM) (Lee et al., 2009), and *Escherichia coli* (Cif, EspF, colibactin) (Azzi-Martin et al., 2019; Fang et al., 2024; Jubelin et al., 2010). *O. tsutsugamushi* Ank1 and Ank6 have been shown to inhibit p65 nuclear translocation by interacting with Lamin A (Evans et al., 2018; Min et al., 2014; Siff et al., 2026). *C. burnetii* incorporates various host cell death-delaying (Cbu0781/AnkG, Cbu1524/CaeA), or host transcriptome modulating (Cbu1314) proteins into host cells (Bisle et al., 2016; Weber et al., 2013, 2016).

These effector proteins manipulate nuclear processes such as histone methylation and acetylation (e.g. RomA methylating H3K14, NUE methylating H3/H4, Rv1988 dimethylating H3R42) (Pennini et al., 2010; Rolando et al., 2013; Yaseen et al., 2015), transcription factor hijacking (e.g. VirE3 (*A. tumefaciens (*Lacroix et al., 2005*)*) or TRPs acting as activators (Wakeel et al., 2011)), and DNA methylation (e.g. Rv2966c or HsdM modifying CpG and non-CpG sites) (Lee et al., 2009; Sharma et al., 2015). Several of these proteins are epidemiologically associated with cancers (gastric, colorectal, or hepatobiliary) presumably due to their influence on chromatin dynamics, transcriptional control, or DNA damage effects (Khan and Khan, 2021; Korgaonkar et al., 2025). To reach the host nucleus, such NMs must first be delivered into host cells through specialized bacterial secretion systems.

Most bacteria employ conserved mechanisms for exporting proteins across their own cytoplasmic membrane, primarily via the Sec and Tat pathways (Jamali et al., 2025), responsible for translocating unfolded and folded proteins, respectively. In Gram-negative bacteria, these exported proteins may remain in the periplasm or be further transported across the outer membrane by additional systems. Gram−positive bacteria, lacking an outer membrane, generally secrete exported proteins directly into the extracellular or host environment. Dedicated secretion systems have evolved with distinct structural and mechanistic features (Blasey et al., 2022; Manisha et al., 2024; Zhou et al., 2024). Capable of transferring both proteins and DNA, the type IV secretion system (T4SS) is used by bacteria like *A. tumefaciens* and *L. pneumophila (*Paillard et al., 2025*;* Roy et al., 2025*)*. The type VI secretion system (T6SS) is present in various Gram-negative bacteria, and required for the secretion of several toxins as well as virulence and fitness factors (Blondel et al., 2025; Matte et al., 2024). This targeted injection allows bacteria to deliver effectors that subvert host immune responses, alter signaling pathways, and promote intracellular survival.

Type II, V, VII, and IX secretion systems typically mediate a two-stage process by first exporting proteins to the periplasm (in Gram-negative bacteria), and then secreting them across the outer membrane. Type II systems, for example, are used to secrete enzymes and toxins that act in the extracellular space or on host cell surfaces (Green and Mecsas, 2016; Naskar et al., 2021). Type III secretion systems span bacterial and host membranes, enabling direct transfer of effector proteins into the target cell (Jia and Zhu, 2025).

However, to directly hijack the command center of eukaryotic host cells, proteins delivered into the cell body must cross an additional barrier: the nuclear membrane. Eukaryotic nuclear transport depends on importins and the nuclear pore complex (NPC). Proteins exceeding ~40–60 kDa require a nuclear localization signal (NLS), a short, basic amino acid sequence rich in lysine (K) and arginine (R). The canonical (classical) NLS system involves importin-α binding to the NLS and presentation to importin-β, which ferries the complex through the NPC. Classical NLSs are categorized as monopartite (single cluster of basic residues, e.g. PKKKRKV) or bipartite (two clusters separated by 10–12 amino acids, e.g. KR[10x]KRRK) (Fontes et al., 2000; Lange et al., 2010). Non-classical variants (PY-NLS, arginine-rich, or hybrid motifs) may bypass importin-α to directly bind importin-β (Lu et al., 2021).

Many bacterial NMs exploit canonical nuclear localization signals to enter the host nucleus, using either classical monopartite or bipartite motifs. Examples include *H. pylori* HP0425 (³KKELLKMSKKR^13^) and UreA (^21^KKRKEK^26^) (Kim et al., 2016; Lee et al., 2015), *L. monocytogenes* LntA (^122^IDAIKRSSEASADTEAFKKIFKEW^144^) (Lebreton et al., 2014), *K. pneumoniae* HsdM (^7^KKAKAKK^13^) (Lee et al., 2009), and *M. tuberculosis* Rv0256c (^473^RRRRPKIKQ^481^) (Bisht et al., 2023). Other NMs, such as *L. pneumophila* RomA and LegAS4 (Li et al., 2013; Rolando et al., 2013), and *C. burnetii* CaeA, AnkG, and Cbu1314 (Bisle et al., 2016; Schafer et al., 2017; Weber et al., 2016), also possess classical or multiple predicted NLSs. In contrast, some effectors like *E. coli* EspF feature atypical non-classical NLS-like regions, while others (e.g. *Shigella* OspF or *Legionella* AnkX) rely on host importins or NLS-bearing partners for nuclear import (Campellone et al., 2004; Jo et al., 2017; Zhao et al., 2019). Mutations or deletions in the NLS typically abolish nuclear localization and reduce pathogen virulence.

The WHO defined the group of ESKAPE pathogens, comprising *E. faecium, S. aureus, K. pneumoniae, A. baumannii, P. aeruginosa*, and Enterobacter species (WHO, 2017). These organisms “escape” antibiotic pressure via multidrug resistance, biofilm formation, and immune evasion, representing the leading cause of nosocomial infections. The majority of these pathogens maintain intracellular lifestyles, enabling survival within epithelial cells, macrophages, hepatocytes, or endothelial cells. Representative examples including *S. aureus*, *K. pneumoniae, A. baumannii*, *P. aeruginosa*, and *E. faecalis* replicate intracellularly *in vitro* and *in vivo*. Our own unpublished data also suggest that *E. faecium* is present inside various human model cell lines (unpublished). Considering the recent update of clinically relevant pathogens (WHO, 2024), a thorough understanding of underlying pathogen-host interactions becomes increasingly urgent.

In summary, NMs represent a convergent evolutionary innovation among plant, animal, and human pathogens, enabling (epigenetic) control of host cells through classical nuclear trafficking mechanisms. Although NMs constitute attractive diagnostic and therapeutic targets, particularly in clinically relevant pathogens such as ESKAPE species, their mechanisms of action remain poorly understood.

To address this emerging topic, our study aimed to identify bacterial cNLS-bearing proteins, which are putative NMs involved in the modulation of host cells during the infection process. cNLS were predicted using an *in silico* analysis, based on NLStradamus.

## Methods

We applied either in-house sequenced or publicly available and annotated genomes for the prediction of canonical NLS with NLStradamus (Nguyen Ba et al., 2009). For prediction, the prediction cutoff was set to 0.6 (default). Initially, the 2 state HMM static was used. A list of all cNLS positive proteins was extracted and categorized into: ABC transporters and related proteins; Other transporters and exporters; DNA and RNA metabolism; Metabolic enzymes; Cell wall, envelope and peptidoglycan-related proteins; Cellular and surface structure proteins; Signal transduction, regulation and stress response; Ribosome, translation and rRNA-related proteins; DNA/genome mobilization and mobile elements; proteins with Domain of unknown function („DUF-domain containing”); and Hypothetical proteins. cNLS were verified using NucPred (Brameier et al., 2007) and NucImport (Mehdi et al., 2011), again setting the respective cutoff to 0.6. For NucImport, values for overall nuclear import and NLS-mediated import were documented separately. For signal peptide prediction of proteins previously annotated to contain a cNLS (NLStradamus), we used SignalP 6.0 (Teufel et al., 2022).

The following bacterial genomes were analyzed in this study:

*S. aureus*: SH1000, EDCC5055, EDCC5464, and EDCC5458 (re-sequenced in-house using Illumina and Oxford Nanopore sequencing and annotated by PGAP (Tatusova et al., 2016)). *E. faecium* (sequenced in house and annotated (Tatusova et al., 2016): V2 (23x1609/ST80), V3 (23x1608/ST117), V5 (6182/ST1299; GenBank: JBNXYM000000000), V6 (7282/ST1299; GenBank: JBNXWZ000000000). One additional strain was used for comparison *E. faecium* LFYP64 (GenBank GCA_902652295.1). *E. faecalis*: V583 (GenBank: GCA_000007785.1), JH2-2 (GenBank: GCA_000479105.1), OG1RF (GenBank: GCA_000172575.2). *A. baumannii*: ATCC19606 (GenBank: GCA_019331655.1), ATCC17978 (GenBank: GCA_013372085.1), AB5075 (GenBank: GCA_000770605.1). *K. pneumoniae*: ATCC43816 (GenBank: GCA_016071735.1), RJF293 (GenBank: GCA_001530015.1), KP1, KP3, KP6 (Neumann et al., 2023). *H. pylori*: J99 (GenBank: GCA_000982695.1), G27 (GenBank: GCA_000021165.1), 26695 (GenBank: GCA_000307795.1). All used datasets are provided as **Supplementary Data S3**.

## Results

### Re-evaluation of known cNLS-bearing proteins using NLStradamus

Analyzing the sequences of known NMs from mammalian pathogens using NLStradamus (Nguyen Ba et al., 2009), we verified various proteins with previously reported NLS (**Table 1**): Cas9 (Acc: Q0P897; *C jejunii* ATCC 700819) (Saha et al., 2020a, b), Omp18 (Acc: A0A7G1HN99*; H. pylori HpKE21*) (Shan et al., 2015), Secreted protein involved in flagellar motility (Acc: A0A238GW57; *H. pylori BCM-300*) (Lee et al., 2012), UreA (Acc: P14916; *H. pylori 26695*) (Konieczna et al., 2012), HsdM (Acc: A0A377WE58; *K. pneumoniae NCTC204) (*Lee et al., 2009*)*, Rv0256c (Acc: P9WI47; *M. tuberculosis* ATCC 25618) (Bisht et al., 2023).

**Table 1:** List of known NMs for which we verified cNLS using NLStradamus (1-6), cNLS could not be verified (7-12, light gray), cNLS were not published but found using NLStradamus (13-18).

Protein/accession	Species	Predicted NLS NLStradamus	Reference originale
Cas9/Q0P897	*C. jejunii*	47 - PRRLARSARKRLARRKARL - 65	(Saha et al., 2020b)
Omp18/A0A7G1HN99	*H. pylori*	51 - PPCFTEPKKPKRK – 63	(Shan et al., 2015)
Secreted protein involved in flagellar motility/A0A238GW57	*H. pylori*	7x repetitive: HKDKKDAKKPE	(Lee et al., 2012)
UreA/P14916	*H. pylori*	21 - KKRKEKG – 27	(Konieczna et al., 2012)
HsdM/A0A377WE58	*K. pneumoniae*	7 - KKAKAK – 12	(Lee et al., 2009)
Rv0256c/P9WI47	*M. tuberculosis*	471 - PQRRRRPKIK – 480	(Bisht et al., 2023)
OmpA and Tnp	*A. baumannii*	Not found	(Choi et al., 2008; Moon et al., 2012)
CdtB, EspF, Tus	*E. coli*	Not found	(Dean et al., 2010; Kaczmarczyk et al., 2010; McSweeney and Dreyfus, 2004)
HP0425, HP0059	*H. pylori*	Not found	(Kim et al., 2016; Kwon et al., 2016)
LntA	*L. monocytogenes*	Not found	(Lebreton et al., 2014)
Rv1988	*M. tuberculosis*	Not found	(Yaseen et al., 2015)
Urease-γ	*P. mirabilis*	Not found	(Grahl et al., 2021)
Cbu0388/Q83ED7	*C. burnettii*	922 - KKPSKKVKIKKSKPKKKK - 939	(Wallqvist et al., 2017; Weber et al., 2016)
Cbu0794/Q83DE4	*C. burnettii*	350 - RRLPPKMRHKAG – 361	
Cbu1314/Q83C21	*C. burnettii*	207 - KTKPAKR – 213	
CT311/O84313	*C. trachomatis*	33 - LRERRKDLHVSGKPSPRYALKKRALEAKKNK – 63	(Lei et al., 2013)
LegAS4/Q5ZUS4	*L. pneumophila*	22 - KLKKKSALQSKFKEQQLNHGSKEHKSKFKFSQRKAKKKGP – 61	(Li et al., 2013)
App/Q8GKS4	*N. meningitidis*	933 - RRRSRRSR – 940	(Khairalla et al., 2015)
SuAT1/D3XPG6	*T. annulata*	140 - KPKKLRKHKPKIKDTDYKARKSKKKS - 165 391 - IAKPKKPRIRRPRKHKSKSETEKVGKPKRKRGRPRKQKPELEEPKRKRGRPKKHK – 445 462 - IDKRKSKLGRPKIK – 475	(Shiels et al., 2004)
Sap11/A0A1Q1NH98	*E. sativa*	96 - KSKKKGSSSKKPDDSKK - 112	(Sugio et al., 2011)
Brg11/Q8XYE3	*R. solanacearum*	31 - RRRPR – 35/185 - RSARARRA – 192/980 - RIRR – 983	(de Lange et al., 2013)
RipAB/A0A7U7JE37	*R. solanacearum*	95 - GKRKRDEETDPNNEADGKKKKKKR – 118	(Zheng et al., 2019)
AvrBs3/P14727	*X. euvesicatoria*	129 - RPPRAKPAPRRR – 140	(Canonne and Rivas, 2012)
XopD/Q8RJQ0	*X. euvesicatoria*	54 - RRKLALAAPKSKPTPKSKPLKG – 75 534 - QKKKKSKWWKKF – 545	(Canonne et al., 2011; Kim et al., 2008)
AvrHah1/B0YIU4	*X. hortorum*	129 - RPPRAKPAPRRR – 140	(Schornack et al., 2008)
AvrXa5/B9VQX6	*X. oryzae*	129 - RPPRAKPAPRRR – 140	(Kay et al., 2007)
AvrXa10/Q56830	*X. oryzae*	128 - RPPRAKPAPRRR – 139	(Romer et al., 2007)
PthXo1/B2SU53	*X. oryzae*	129 - RPPRAKPAPRRR – 140	(Mak et al., 2012)

Verified NLS in NM from plant pathogens and protozoa (19-28).

Published NLSs of *A. baumannii* (OmpA and Tnp), *E. coli* (CdtB, EspF, Tus), *H. pylori (*HP0425, HP0059), *L. monocytogenes* (LntA), *M. tuberculosis* (Rv1988), and *P. mirabilis* (Urease-γ) could not be verified (Bierne and Cossart, 2012; Khan and Khan, 2021; Korgaonkar et al., 2025) (**Table 1**).

In addition, we predicted NLS for NMs without hitherto known NLS (**Table 1**). These include *C. burnettii* RSA 493 Cbu0388 (Acc: Q83ED7), Cbu0794 (Acc: Q83DE4), Cbu1314 (Acc: Q83C21) (Wallqvist et al., 2017; Weber et al., 2016), the hypothetical protein CT311 (ACC: O84313; *C. trachomatis* D/UW-3/Cx) (Lei et al., 2013), LegAS4 (Acc: Q5ZUS4; *L. pneumophila* Philadelphia 1/ATCC 33152) (Li et al., 2013) and App (Acc: Q8GKS4; *N. meningitidis* H44/76) (Khairalla et al., 2015).

For SuAT1 from the apicomplexan *T. annulata* (Acc: D3XPG6) affecting protozoa and human cells (Shiels et al., 2004), we could also verify a NLS (**Table 1**).

Checking NMs from plant pathogens, we retrieved cNLS for Sap11 (Acc:A0A1Q1NH98 *‘Eruca sativa’ phytoplasma* QU-RO1) (Sugio et al., 2011), Brg11 (Acc: Q8XYE3; *Ralstonia solanacearum* ATCC BAA-1114/GMI1000) (de Lange et al., 2013), RipAB (Acc: A0A7U7JE37; *R. solanacearum* IPO1609) (Zheng et al., 2019), AvrBs3 (Acc: P14727; *Xanthomonas euvesicatoria* 71-21) (Canonne and Rivas, 2012), AvrHah1 (Acc: B0YIU4; *X. hortorum*) (Schornack et al., 2008), AvrXa5 (Acc: B9VQX6; *X. oryzae* pv. oryzae JXOIII) (Kay et al., 2007), AvrXa10 (Acc: Q56830; *X. oryzae pv. oryzae* PXO86) (Romer et al., 2007), PthXo1 (Acc: B2SU53; *X. oryzae pv. oryzae* PXO99A) (Mak et al., 2012), and XopD (Acc: Q8RJQ0; *X. euvesicatoria* 75-3) (Canonne et al., 2011; Kim et al., 2008) (**Table 1**).

Taken together, these results show that we can predict and/or verify cNLS in a variety of known NMs from various plant and mammalian pathogens. This encouraged us to apply the workflow to a selection of ESKAPE pathogens frequently used to investigate pathogen-host-interactions, thereby enabling us to unveil possible novel actors that modulate the pathogen-host interface.

### Prediction of cNLS-bearing proteins in *S. aureus*

In a previous study, we revealed differential host responses of different human model cell lines to intracellular infection with the four *S. aureus* isolates SH1000, EDCC5055, EDCC5464, and EDCC5458. Performing transmission electron microscopy, we frequently observed bacteria in a nuclear membrane-proximal region and partially also within endonuclear compartments (Walter et al., 2026). The biological relevance of the latter remains to be thoroughly characterized. However, similar observations were made in arthropods, amoeba, and marine invertebrates, showing that bacteria can hijack the host cell’s nucleus (Porras et al., 2024; Schulz and Horn, 2015). In mammalian cells, such nuclear-proximal or intranuclear localization has been shown for *Rickettsia* spp. and *O. tsutsugamushi* (formerly *R. tsutsugamushi*) (Burgdorfer et al., 1968; Rikihisa and Ito, 1979). Such localization in our infection models thus prompted us to screen these *S. aureus* isolates for the presence of cNLS-bearing proteins, as nuclear proximity may facilitate the ability of NMs to pass the nuclear membrane.

We identified 119 proteins for SH1000, 132 for EDCC5055, 126 for EDCC5464, and 140 for EDCC5458 using the 2-state HMM model. Using the 4-state HMM model on strain EDCC5458 (previously most identified cNLS-positive proteins), we identified 22 additional proteins, whereas 2 proteins were uniquely detected by the 2-state static model. All further analyses were therefore based on predictions from the 2-state model. A complete list of the identified proteins is provided in **Supplementary Table 1**.

**Figure 1:**
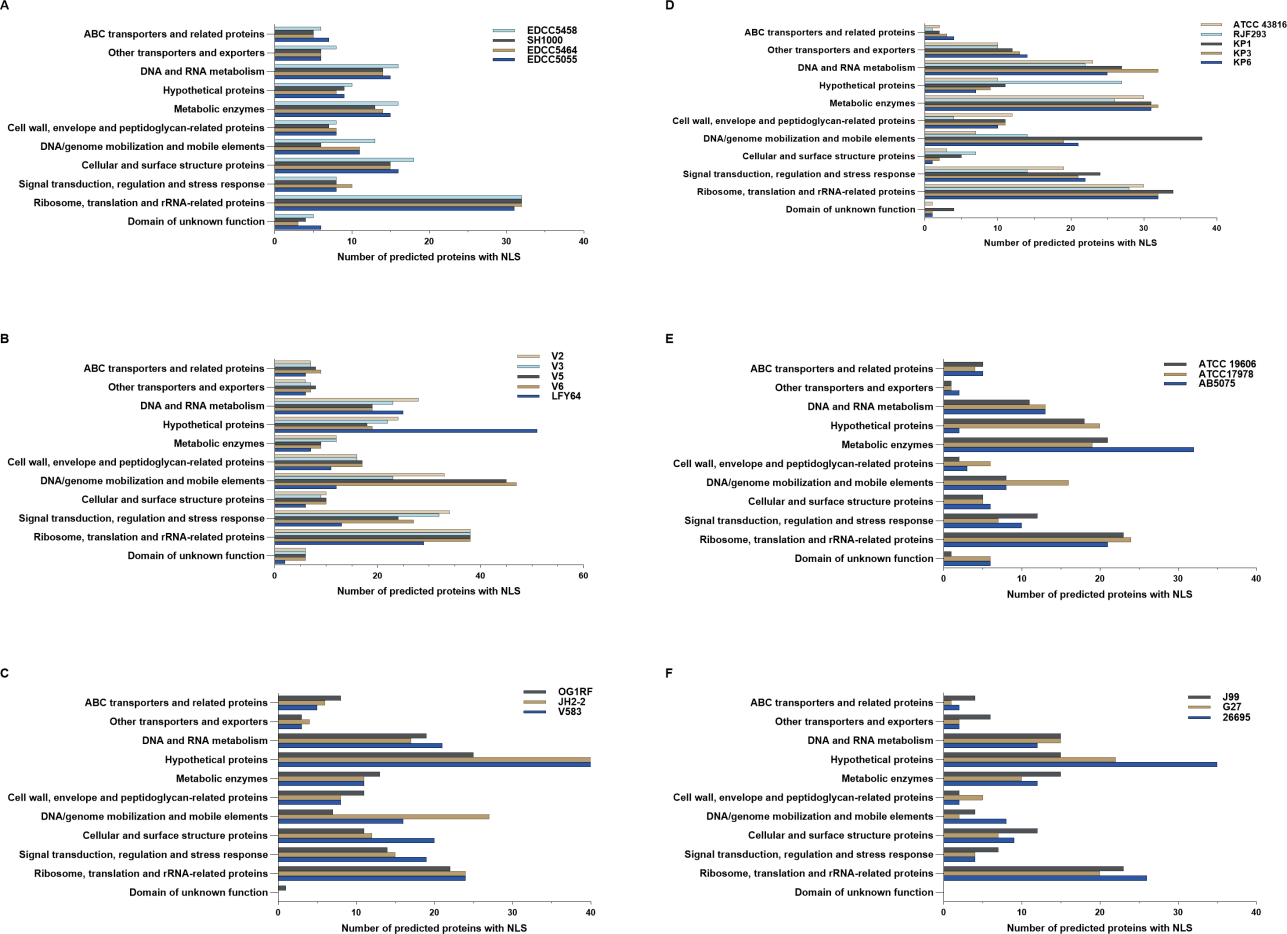
Identified and grouped candidate Nucleomodulins: **(A)** *S. aureus*, **(B)** *E. faecium*, **(C)** *E. faecalis*, **(D)** *K. pneumoniae*, **(E)** *A. baumannii*, and **(F)** *H. pylori*. The bars indicate the number of individual candidates per category.

Classification of all cNLS-positive proteins into functional categories revealed no major qualitative differences among the four *S. aureus* isolates (**Figure 1A**). Seven proteins occurred as duplicates with identical annotations; four proteins annotated as hypothetical proteins, 10 candidates were found, mostly with unique sequences.

We identified 140 cNLS-positive proteins in EDCC5458 using NLStradamus, covering most of the corresponding proteins in the other isolates (with some missing and a few additional ones found). These proteins were further analyzed with the prediction tool NucPred.

NucPred equally predicted NLS for all 140 proteins using default settings. Values above 0.6 for 40 proteins are listed in **Supplementary Table 1**.

Thereupon, the previously identified 140 cNLS-positive proteins were further analyzed using NucImport, which predicts not only the presence of NLS but also the overall likelihood of nuclear import by considering interaction with known importins. Consistent with the previous analysis, a threshold of 0.6 was applied, with initial evaluation focusing exclusively on the predicted nuclear import probability. The NucImport results were subsequently compared with the 40 proteins previously identified by NucPred. Only proteins that achieved a score greater than 0.6 in both prediction tools were retained, yielding in a final list of 24 proteins (summarized in **Supplementary Table 1**, marked in red).

Finally, the probability of NLS presence predicted by NucImport was examined by applying the same threshold to these 24 proteins (**Supplementary Table 1**). The resulting set of 13 proteins (**Supplementary Table 1**, red and bolded) was consistently identified by three independent prediction tools as having a high likelihood of carrying an NLS and undergoing nuclear import, thus representing strong candidates for putative NMs (**Table 2**).

**Table 2:** List of candidate *S. aureus* nucleomodulins.

Protein	NucPred	NucImport (I)	NucImp(N)	SignalP	Reference
SasC (000352_309)	0.79	0.949	0.849	X	(Schroeder et al., 2009)
DNA translocase FtsK (000367_324)	1.00	0.993	0.814		(Chan et al., 2022; Veiga et al., 2023)
30S Rib S21 (000533_489)	0.80	0.998	0.939		
50S-Rib_L34 (02131_2059)	0.770	0.989	0.644		
PVL family (000640_596)	0.81	0.986	0.602		(Adler et al., 2006; Genestier et al., 2005)
Helix-turn-Helix containing protein (000673_629)	0.790	0.985	0.690		(Chu et al., 2022; Fan et al., 2015; Morrison et al., 2012)
Ebh (000760_715)	0.780	0.962	0.761	X	(Bui et al., 2015; Cheng et al., 2014; Clarke et al., 2002; Kuroda et al., 2008)
FtsQ/DivB (001018_973)	0.980	0.972	0.859		(Bottomley et al., 2014)
TypeIR_HsdR (001998_1928)	0.72	0.813	0.813		(Szczelkun, 2000; Westra et al., 2012)
DEAH/DEAD box helicase (002784_2703)	0.74	0.962	0.737		
DUF4887-containing (001411_1362)	0.820	0.781	0.655		
DUF1450-containing (001593_1544)	0.870	0.995	0.837		
Hypothetical (001503_1454)	0.690	0.822	0.637	X	

Cut-off values for NucPred and NucImport are provided. Predicted signal peptides are indicated (X). Pgaptmp loci in brackets ensure unambiguous assignment to the respective gene. Proteins are marked in red/bold in **Supplementary Table 1**.

The presence of a signal peptide, indicating the secretion of the respective protein, can serve as an independent criterion to identify NMs. Using SignalP (Teufel et al., 2022), we again scanned the 140 cNLS-positive proteins from EDCC5458 and obtained a shortlist of 20 proteins for which signal peptides were predicted (**Supplementary Table 1**).

While no hits were detected within the DNA-binding protein groups (DNA & RNA metabolism, DNA/genome mobilization and mobile elements), several were identified in the categories cellular and surface structure proteins (8/18), ABC transporters and related proteins (3/6), and Hypothetical proteins (3/10).

In addition to signal peptide prediction based on the cNLS-shortlist, we screened the complete genome of isolate EDCC5458 using SignalP. In total, 179 proteins were predicted to harbor a signal peptide (**Supplementary Table 2**), some of which could represent additional NMs lacking a predictable canonical NLS.

The identified candidate NMs can be grouped according to their known or predicted functions, with DNA/RNA-binding proteins showing the highest relevance for putative nuclear modulation. This classification also reflects current knowledge on bacterial NMs, which frequently target host chromatin structure, transcriptional regulation, and other nuclear processes through DNA- or RNA-associated activities. In the case of *S. aureus*, the functional spectrum of the identified candidates ranges from proteins involved in chromosome segregation, cell division, and RNA metabolism to toxins, surface-associated proteins, and proteins with unknown function.

### Cell division and DNA/RNA binding

FtsK belongs to the FtsK/SpoIIIE family of DNA-binding proteins, regulating chromosome segregation and daughter cell separation in Gram-positive and -negative bacteria cell cycle. In *S. aureus*, FtsK has been shown to coordinate chromosome segregation with daughter cell separation, linking DNA processing to cell division (Chan et al., 2022; Veiga et al., 2023). Additionally, the FtsK ATPase domain has been implicated in regulating secretion of proteins such as ESAT-6 or the related *S. aureus* proteins EsxA and EsxB (Aly et al., 2017; Burts et al., 2005; Pallen, 2002). Likewise, FtsQ/DivIB proteins are involved in cell division and septum formation in Gram-positive and Gram-negative bacteria. In *S. aureus*, DivIB has further been described as a peptidoglycan-binding protein required for a morphological checkpoint during cell division (Bottomley et al., 2014). Helix-turn-helix proteins bind to DNA and RNA and frequently act as transcription factors in eukaryotes, archaea, and prokaryotes, including *S. aureus* (e.g. virulence factors SaeR or SasA). Recent RNA interactome data additionally indicate that proteins with helix-turn-helix DNA-binding domains can display post-transcriptional RNA-binding functions in MRSA, supporting a broader regulatory potential (Chu et al., 2022; Fan et al., 2015; Morrison et al., 2012). Type I restriction endonuclease R (HsdR) typically degrades non-self DNA (Szczelkun, 2000; Westra et al., 2012). DEAD/DEAH box helicases regulate bacterial RNA metabolism and quorum sensing. In *S. aureus*, CshA contributes to quorum sensing control and is involved in RNA turnover, highlighting its role in post-transcriptional regulation (Oun et al., 2013; Redder and Linder, 2012).

### Ribosomal proteins

No additional information is available for the *S. aureus* homologue, while 30S ribosomal protein S21 produced by *Lactobacillus sakei* harbors antimicrobial activity (de Carvalho et al., 2010). The 50S ribosomal protein L34 (rpmH), part of the large ribosome subunit in *E. coli*, has no further known function in its *S. aureus* homolog rpmH (Hansen et al., 1982). Nevertheless, ribosomal proteins were repeatedly identified among candidate proteins in different species analyzed in this study, and ribosomal proteins in general are increasingly recognized to display moonlighting functions beyond their canonical role in translation.

### Toxins

PVL (Panton-Valentine Leukocidin) is a secreted cytotoxin that causes lytic host cell death and tissue necrosis (e.g. necrotizing pneumonia), or directly target mitochondria in human neutrophils to induce Bax-independent apoptosis, supporting a role in intracellular host cell manipulation beyond membrane damage alone (Adler et al., 2006; Genestier et al., 2005).

### Surface proteins (biofilm/clumping)

The LPXTG-anchored repetitive surface protein SasC mediates *S. aureus* cell clumping and biofilm formation (Schroeder et al., 2009). Similarly, Ebh is a cell wall bound protein associated with hyperosmotic pressure tolerance, fibronectin/extracellular matrix binding, clumping, and cell size control (Cheng et al., 2014; Clarke et al., 2002; Kuroda et al., 2008). Ebh has also been linked to the small-colony variant (SCV) phenotype, which is often associated with intracellular lifestyle and relapsing disease (Bui et al., 2015).

### Others (function unknown)

Hypothetical proteins and those with domains of unknown function (DUF-domain containing) have no reported function. At the same time, proteins of unknown function remain of particular interest in the nucleomodulin context, as several bacterial NMs were initially annotated as hypothetical proteins before functional characterization.

Taken together, the predictions obtained from NLStradamus, NucPred, NucImport (“Import”) and SignalP allow the identification of several proteins that represent strong candidates for putative NMs. Three proteins fulfill all defined criteria, showing prediction scores above the 0.6 threshold in NLStradamus, NucPred and NucImport (“Import”) and additionally containing a predicted signal peptide: SasC, Ebh, and hypothetical protein (001503-1454).

### Prediction of cNLS-bearing proteins in *E. faecium and E. faecalis*

The Gram-positive commensals *E. faecium* and *E. faecalis* are the leading enterococcus species in clinical settings, particularly those harboring vancomycin resistance (VREfm/fc). Among them, the ST80, ST117, and the recently established ST1299 are dominant *VREfm* sequence types in south-eastern Germany (Rath et al., 2024, 2023). Information on intracellular lifestyles and pathogen-host-interactions are scarce for *E. fa*e*cium*. Three case reports hint toward intracellular survival by identification of SCVs (Egido et al., 2016; Gröbner et al., 2012; Höring et al., 2019). Own data revealed uptake of *E. faecium* by different cell lines and induction of differential gene expression in U937 monocytes (unpublished data). For *E. faecalis*, various studies reported uptake and intracellular lifestyle in different human host cells (Baldassarri et al., 2005; da Silva et al., 2022; Horsley et al., 2013; Sussmuth et al., 2000; Wells et al., 2000).

We analyzed four locally collected and sequenced isolates (V2/ST80, V3/ST117, V5/ST1299, V6/1299) and one additional isolate from the US (LFYP64), using the same criteria as described for *S. aureus*. For each isolate, we identified the following numbers of cNLS-positive proteins: V2: 214, V3: 195, V5: 202, V6: 208, LFYP64: 168, which were sorted into protein groups (**Figure 1B**). A complete list of all proteins is provided as **Supplementary Table 1**. Notably, for LFYP64, 52 hypothetical proteins and only 12 for DNA/genome mobilization and mobile elements were annotated, compared to 18–24 and 23–47 proteins, respectively, in the other isolates.

The strain V2 was selected as a representative isolate, and its proteins predicted as cNLS-positive by NLStradamus (214), NucPred (57) and NucImport (Import: 34/NLS: 14) are listed in **Table 3** (cutoff 0.6). No *E. faecium* protein was positive for all NLS criteria and SignalP. Only the Serine hydrolase domain-containing protein signal peptide was positive. For a total of 132 proteins a signal peptide was predicted without previously screening for cNLS (**Supplementary Data S2**). Again, we grouped the putative *E. faecium* NMs as before and listed only those proteins for which a role as a NM is plausible based on their described function.

**Table 3:** List of candidate *E. faecium* nucleomodulins.

Protein	NucPred	NucImport (I)	NucImp (N)	SignalP	Reference
group II intron reverse transcriptase/maturase (000987_969/002665_2593)	0.81	0.857	0.611		(Zhao and Pyle, 2017)
tyrosine-type recombinase/integrase (000372_369)	0.65	0.832	0.678		(Zhang et al., 2023)
IS3-like element ISEfa8 family transposase (001264_1216/002110_2042)	0.80	0.955	0.701		(Zhang et al., 2023)
ABC-F type ribosomal protection protein Msr(C) (001097_1079)	0.83	0.94	0.662		(Hayes et al., 2005).
30S ribosomal protein S18 (000929_923)	0.66	0.953	0.602		
30S ribosomal protein S21 (001824_1760)	0.84	0.992	0.698		
50S ribosomal protein L34 (000938_932)	0.85	0.991	0.671		
50S ribosomal protein L35 (000701_695)	0.62	0.957	0.644		
cell division site-positioning protein MapZ family (002782_2709)	0.76	0.96	0.781		(Feng et al., 2019; Fleurie et al., 2014; Manuse et al., 2016)
glycoside hydrolase family 1 protein (000286_283)	0.96	0.883	0.605		(Bautista-Cruz et al., 2024)
fibronectin-binding protein EfbA (02503_2434)	0.86	0.958	0.781		(Shimosaka et al., 2025) (Torelli et al., 2012) (Singh et al., 2015)
RsiV family protein (001731_1670)	0.74	0.91	0.793		(Hastie et al., 2016; Pannullo and Ellermeier, 2022; Parthasarathy et al., 2021)
hypothetical protein (1655_1594)	0.72	0.926	0.74		
Serine hydrolase domain-containing protein (002956_2864)	0.74	0.752	(0.266)	X	

Cut-off values for NucPred and NucImport are provided and predicted signal peptides are indicated (X). Pgaptmp loci in brackets are provided to ensure unambiguous assignment to the respective gene. Proteins are marked in red/bold in **Supplementary Table 1**.

Again, the identified candidate NMs can be grouped by their known or predicted functions and relevance for nuclear modulation. In the case of *E. faecium*, the functional spectrum ranges from genome plasticity and cell division factors to ribosomal proteins and virulence-associated proteins.

### Cell division and DNA/RNA binding

For *E. faecium*, we identified three DNA/RNA-binding and modulating enzymes. Group II intron reverse transcriptase/maturases regulate RNA maturation and enable retro-transposition of introns/mobile genetic elements into (specific) DNA sites (Zhao and Pyle, 2017). Tyrosine-type recombinase/integrases are site-specific recombinases mediating horizontal gene transfer (Zhang et al., 2023). IS3-like element ISEfa8 family transposases regulate mobile elements, and have been associated with prolonged *E. faecium* and *E. faecalis* bacteremia (Udaondo et al., 2022). In addition, MapZ is involved in the regulation of cell division processes in various species, including enterococci (Feng et al., 2019; Fleurie et al., 2014; Manuse et al., 2016). RsiV (anti-sigma factor) has been described as bacterial lysozyme-binding factor that mediates resistance via mechanisms involving the transmembrane protease Eep in various species, including enterococci (Hastie et al., 2016; Pannullo and Ellermeier, 2022; Parthasarathy et al., 2021).

### Ribosomal proteins

The 30S ribosomal protein subunits S18 and S21 and the 50S ribosomal subunits L34 and L35 were identified. Both S21 and L34 were also present among the *S. aureus* proteins. The 30S ribosomal protein S18 is highly conserved, with the only reported function to regulate ribosome stability (Agalarov et al., 2000). The 50S ribosomal protein L35 is upregulated (along with most ribosomal subunits) upon *S. suis* infection of U937 monocytes, likely reflecting adaptation to intracellular survival (Prangsuwan et al., 2025).

### Additional candidate proteins

Msr(C) confers resistance to macrolide and lincosamide antimicrobials (Hayes et al., 2005). Glycoside hydrolase family 1 (GH1) proteins mediate hydrolysis of carbohydrates across diverse organisms (Bautista-Cruz et al., 2024). The fibronectin-binding protein EfbA has been described as a virulence factor of *E. faecalis* which promotes invasion of human pancreatic duct epithelial cells (Shimosaka et al., 2025), urinary tract infection in mice (Torelli et al., 2012), and endocarditis in a rat model (Singh et al., 2015). For *E. faecium*, diverse serine hydrolases, cleaving a variety of substrates, including antibiotics, have been verified on protein level (Grunnvag et al., 2024). No detailed information is available for serine hydrolase domain-containing protein.

Next, we analyzed three *E. faecalis* strains regarding NLS-positive proteins: V583 (199), JH2-2 (166), OG1RF (134). All proteins were grouped as shown in **Figure 1C**. V583 was selected as the representative strain. For this isolate, 11 proteins were positive in NLStradamus, NucPred and Nucimport as shown in **Table 4**. No *E. faecalis* protein met all NLS criteria and was positive SignalP. In total, the presence of a signal peptide was predicted for a total of 209 proteins without previous screening for cNLS (**Supplementary Data S2**). Grouping of proteins was performed as described above, naming only proteins with plausible functionality.

**Table 4:** List of candidate *E. faecalis* nucleomodulins.

Protein	NucPred	NucImport (I)	NucImp (N)	Reference
ATP-dependent RNA helicase, DEAD/DEAH box family (81168.1)	0.76	0.991	0.748	
DNA-directed RNA polymerase, omega (82805.1)	0.63	0.98	0.825	(Cheng et al., 2023; Mao et al., 2018; Mathew and Chatterji, 2006)
group II intron reverse transcriptase maturase (82051.1)	0.79	0.899	0.70	(Park et al., 2025, 2022)
ParB-like nuclease domain protein (82013.1)	0.64	0.876	0.634	(Osorio-Valeriano et al., 2021, 2019)
transcriptional regulator, PSR protein (81356.1)	0.77	0.829	0.762	(Kang et al., 2009; Shen et al., 2006)
site-specific recombinase, phage integrase family (81984.1)	0.77	0.71	0.632	(Groth and Calos, 2004)
ribosomal protein L20 (80724.1)	0.61	0.988	0.654	
ribosomal protein L34 (82998.1)	0.85	0.99	0.64	
ribosomal protein S21 (82135.1)	0.84	0.998	0.932	
peptidase, M23/M37 family (82044.1)	0.86	0.99	0.80	(Razew et al., 2022)
hypothetical protein EF_3146	0.65	0.944	0.637	

Cut-off values for NucPred and NucImport are provided. Loci in brackets are provided to ensure unambiguous assignment to the respective gene. Proteins are marked in red/bold in **Supplementary Table 1**.

In the case of *E. faecalis*, the functional spectrum of putative NM ranges from RNA metabolism and genome stability factors to ribosomal proteins and cell wall hydrolases.

### DNA/RNA binding and genome stability

RNA polymerase (RNAP) is a multimeric complex, which requires the omega subunit for stability. Omega has also been associated with bacterial biofilm formation, growth, stress, and stringent response, even in facultative and obligate intracellular bacterial pathogens (Cheng et al., 2023; Mao et al., 2018; Mathew and Chatterji, 2006). Group II intron reverse transcriptase maturase mediates double-strand break repair (Park et al., 2025, 2022). ParB regulates DNA segregation during cell division (Osorio-Valeriano et al., 2021, 2019). For ATP-dependent RNA helicase, DEAD/DEAH box family 81168.1, no detailed information is available.

### Ribosomal proteins

Three ribosomal proteins have been identified: L20, L34 and S21. L34 was also present in *S. aureus* and *E. faecium*, S21 in *E. faecium* only.

### Additional candidate proteins

The family of zinc-dependent M23/M37 peptidoglycan hydrolases regulates cell division and bacterial cell wall integrity in Gram-positive and -negative organisms (Razew et al., 2022). Phosphate Starving Response (PSR) protein, a transcription factor present in different species, regulates e.g. the type III secretion system in *P. aeruginosa*, depending on the presence of long-chain fatty acids (Kang et al., 2009; Shen et al., 2006). The site-specific integrase refers to a bacteriophage-encoded integrase (Groth and Calos, 2004).

### Prediction of cNLS-bearing proteins in *K. pneumoniae and A. baumannii*

After analyzing two Gram-positive bacterial species, we additionally screened two Gram-negative species. Accordingly, *K. pneumoniae* represents a Gram-negative ESKAPE pathogen exhibiting intracellular lifestyles in a variety of professional and non-professional phagocytic host cell types (Chang et al., 2020; Cortes et al., 2002; Oelschlaeger and Tall, 1997; Sahly et al., 2000). Although commonly designated an extracellular pathogen, several studies reported the capability of *A. baumannii* to actively and passively invade phagocytic and non-phagocytic host cells (Neumann et al., 2023).

Across five *K. pneumoniae* strains used (**Figure 1D**), we identified cNLS-positive proteins: ATCC43816 (147), RJF293 (153) KP1 (199), KP3 (175), KP6 (168) and selected KP1 as representative isolate (**Supplementary Table 1**). NLS positive proteins (cutoff >0.6) are listed in **Table 5**, none was SignalP-positive. In total, a signal peptide was predicted for 618 proteins without additional screening for cNLS (**Supplementary Data S2**).

**Table 5:** List of candidate *K. pneumoniae* nucleomodulins.

Protein	NucPred	NucImport (I)	NucImp (N)	Reference
PTS beta-glucoside transporter subunit IIABC (000883_875)	0.72	0.971	0.847	(McCoy et al., 2015)
ATP-dependent RNA helicase RhlE (004234_4125)	0.61	0.992	0.706	(Cartier et al., 2010; Redder and Linder, 2012)
ATP-dependent RNA helicase SrmB (001310_1294)	0.66	0.993	0.762	(Cartier et al., 2010; Redder and Linder, 2012)
Exodeoxyribonuclease VII large subunit (001194_1178)	0.65	0.937	0.605	(Poleszak et al., 2012)
ribonuclease E (004484_4371)	0.78	0.979	0.666	(Mackie, 2013)
Polynucleotide adenylyltransferase PcnB (003490_3406)	0.63	0.847	0.759	(Masters et al., 1993, 1990; O'Hara et al., 1995)
NtaA/DmoA family FMN-dependent monooxygenase (005273_5160)	0.91	0.971	0.613	(Ellis, 2010)
Cell division protein DamX (002198_2157)	0.91	0.987	0.841	(Gerding et al., 2009; Khandige et al., 2016; Lopez-Garrido and Casadesus, 2010)
IS66-like element ISKpn24 family transposase (000204_204/000274_274)	0.71	0.972	0.774	(Miller et al., 2021)
ISNCY family transposase (000232_232)	0.84	0.951	0.671	(Miller et al., 2021)
ISNCY-like element ISKpn21 family transposase (000228_228/001056_1046/005088_4975)	0.77	0.846	0.623	(Miller et al., 2021)
Phage tail protein (001255_1239)	0.74	0.962	0.609	(Klein-Sousa et al., 2025; Prevelige and Fane, 2012)
Phage virion morphogenesis protein (001891_1858)	0.74	0.862	0.629	
Der GTPase-activating protein YihI (002635_2587)	0.79	0.989	0.608	(Hwang and Inouye, 2010)
Hypothetical protein (000434_434)	0.60	0.979	0.812	
DNA translocase FtsK (004329_4220)	0.78	0.985	(0,48)	(Bigot et al., 2007)

Cut-off values for NucPred and NucImport are provided. Pgaptmp loci in brackets are provided to ensure unambiguous assignment to the respective gene. Proteins are marked in red/bold in **Supplementary Table 1**. NucImp value for FtsK is below 0.6.

For *K. pneumoniae* the identified candidate NMs can be grouped into sugar transporters and RNA helicases to DNA repair enzymes, phage proteins, and cell division factors.

### DNA processing, repair, and genome mobility

Exonuclease VII has been best characterized in *E. coli* and is involved in DNA repair and recombination (Poleszak et al., 2012). The large subunit XseA exhibits caspase-like activity, including the caspase-recognition motif YVAD, and triggers apoptosis-like DNA fragmentation upon overexpression. Cytotoxicity is counteracted by the small subunit XseB (Jung et al., 2015). DamX regulates cell division and morphology e.g. in *E. coli* and *S. enterica*, and controls switching of *E. coli* (UPEC) cell morphology during urinary tract infection (Gerding et al., 2009; Khandige et al., 2016; Lopez-Garrido and Casadesus, 2010). Transposases (IS66-like or ISNCY) regulate mobile genetic elements in bacterial genomes and plasmids (Miller et al., 2021). FtsK was included in the table as it was also identified in *S. aureus* (**Table 1**). It regulates cell division and protein secretion.

### Sugar transport and RNA metabolism

PTS SU IIABS is part of the phosphotransferase system (PTS) required for sugar uptake via bacterial membranes (McCoy et al., 2015). The ATP-dependent helicases RhlE and SrmB belong to the family of RNA-binding DEAD-box helicases (Cartier et al., 2010; Redder and Linder, 2012). PcnB is involved in RNA polyadenylation in various species and regulates RNA-RNA interactions or plasmid stability and copy number (Masters et al., 1993, 1990; O'Hara et al., 1995). Ribonuclease E is part of the RNA degradosome complex (Mackie, 2013).

### Additional candidate proteins

NtaA/DmoA (Nitrilotriacetate/Dimethylsulfide Monooxygenase) belongs to the family of two-component flavin mononucleotide (FMN)-dependent monooxygenases, which catalyse oxidation in various cellular reactions (Ellis, 2010). Phage tail and virion morphogenesis proteins regulate bacteriophage life cycles (Klein-Sousa et al., 2025; Prevelige and Fane, 2012). YihI is a GTPase-activating protein (GAP) identified in *E. coli*, regulating 50S ribosome maturation in conjunction with Der (Hwang and Inouye, 2010). GTPase-regulating proteins are partially highly conserved among bacteria, archaea, and eukarya, which could allow regulation of eukaryotic processes by such bacterial enzymes (Caldon and March, 2003).

In the three *A. baumannii* strains NLStradamus identified cNLS-positive proteins as follows: ATCC17978 (121), ATCC19606 (107) and AB5075 (108) (**Figure 1E**). ATCC17978 was selected as a representative isolate (**Supplementary Table 1**). NLS positive proteins (cutoff >0.6) are listed in **Table 6**, none was SignalP positive. Overall, a signal peptide was predicted for 454 proteins, without previously screening for cNLS (**Supplementary Data S1**).

**Table 6:** List of candidate *A. baumannii* nucleomodulins.

Protein	NucPred	NucImport (I)	NucImp (N)	Reference
polynucleotide adenylyltransferase PcnB (22832.1)	0.71	0.935	0.847	(Masters et al., 1993, 1990; O'Hara et al., 1995)
Rne/Rng family ribonuclease (22666.1)	0.77	0.988	0.719	(Li and Deutscher, 2004)
SPOR domain-containing protein (25015.1)	0.74	0.89	0.745	(Yahashiri et al., 2017)
IS5 family transposase (22484.1/22551.1)	0.75	0.969	0.696	(Miller et al., 2021)
IS5-like element ISAba12 family transposase 24729.1)	0.78	0.96	0.618	
transposase (22499.1/25514.1)	0.97	0.9	0.673	
transposase (25496.1)	0.98	0.903	0.671	
minor capsid protein (23309.1)	0.64	0.891	0.633	
50S ribosomal protein L34 (25721.1)	0.72	0.908	0.611	(Hansen et al., 1982)
YdaU family protein (23295.1)	0.73	0.894	0.668	
YdaU family protein (24242.1)	0.76	0.925	0.691	

Cut-off values for NucPred and NucImport are provided. Loci in brackets are provided to ensure unambiguous assignment to the respective gene. Proteins are marked in red/bold in **Supplementary Table 1**.

For *A. baumannii*, the functional spectrum ranges from RNA metabolism and genome plasticity factors to cell division proteins and ribosomal components.

As for *K. pneumoniae*, PcnB, which regulates RNA polyadenylation and plasmid stability was identified (Masters et al., 1993, 1990; O'Hara et al., 1995). Ribonucleases (e.g. Rne/Rng family ribonucleases) mediate RNA stability (Li and Deutscher, 2004). Transposases, including IS5/IS5-like, regulate the integration and mobility of genetic elements in bacterial genomes and plasmids (Miller et al., 2021). SPOR domain-containing proteins bind to peptidoglycan and regulate cell division and envelope integrity (Yahashiri et al., 2017). As for *E. faecium* and *E. faecalis*, the 50S ribosomal protein L34 (RpmH) was identified, which regulates core ribosome stability. For YdaU proteins (e.g. UniProt: P76065), no known function has been reported.

### Prediction of cNLS-bearing proteins in *H. pylori*

While not accounted for as ESKAPE pathogen, *H. pylori* is a highly prevalent causative pathogen of various gastric diseases and part of the updated WHO priority list of pathogenic bacteria (WHO, 2024). As for *H. pylori* some NMs were already described (Korgaonkar et al., 2025), we selected three strains and predicted putative NMs (**Figure 1F**): HPy_26695: 112, HPy_G27: 88, and Hpy_J99: 103. For the representative isolate HPy 26695, the identified proteins were grouped according to the above terms, and are listed in **Supplementary Table 1**. Four proteins matching the criteria NLS cutoff >0.6 and SignalP positive are listed in **Table 7**. In total, a signal peptide was predicted for 209 proteins without previously screening for cNLS (**Supplementary Data S2**).

**Table 7:** List of candidate *H. pylori* nucleomodulins.

Protein	NucPred	NucImport (I)	NucImp (N)	SignalP	Reference
RNA polymerase sigma factor RpoD (41307.1)	0.70	0.972	0.793		(Solnick et al., 1997)
transcription-repair coupling factor (42764.1)	0.66	0.843	0.843		(Srivastava and Darst, 2011)
hypothetical protein C694_02610	0.78	0.914	0.765	X	
hypothetical protein C694_06415	0.67	0.996	0.841		

Cut-off values for NucPred and NucImport are provided and predicted signal peptides are indicated (X). Loci in brackets are provided to ensure unambiguous assignment to the respective gene. Proteins are marked in red/bold in **Supplementary Table 1**.

While no functional data are available for the hypothetical proteins, RpoD is a highly divergent transcription factor in *H. pylori* and other Gram-negative bacteria, active during exponential growth (Solnick et al., 1997). The transcription-repair coupling factor (TRCF) interacts with RNAP and corrects errors/lesions on DNA template during transcription (Srivastava and Darst, 2011). *H. pylori* UreA, previously identified as a nucleomodulin (Lee et al., 2012) (see **Table 1**), is not listed in the **Table 7** as it was detected only by NLStradamus. The NMs HP0425 and HP0059 (**Table 1**) were identified to harbor DNAse I like activity and were not identified by our approach (Kim et al., 2016; Kwon et al., 2016). “Secreted protein involved in flagellar motility” (**Table 1**) corresponding to “putative secreted motility protein 42683.1” was not positive for NucPred and NucImport, hence not listed in **Table 7**.

## Discussion

An increasing number of bacteria emanate as facultative or obligate intracellular pathogens, that modulate their host cells to generate a niche protected from the host recognition and immune or antimicrobial control.

In this study, we performed an *in silico* prediction of nucleomodulins encoded by *S. aureus, E. faecium, E. faecalis, K. pneumoniae, A. baumannii*, and *H. pylori* using three freely available tools. We initially used NLStradamus, which was trained based on a curated set of yeast protein NLS, to predict canonical NLS. Thus, any other ncNLS-bearing proteins are not detected. In addition to NLStradamus, we used NucPred and NucImport. NucPred was trained using information about nuclear/non-nuclear localization of proteins, without considering known NLS (Brameier et al., 2007). NucImport predicts nuclear localization based on NLS sequences and importin interaction (Mehdi et al., 2011). In the present study, we used a selection of bacterial genomes, which may not entirely reflect the heterogeneity of the respective species. Our approach also assumes that putative bacterial NLS are structured similarly to eukaryotic ones. This has to be confirmed by integrating all currently known bacterial NMs (with and without NLS sequences).

A similar workflow was recently used to predict NMs in *F. nucleatum*, identifying 330 out of 2204 proteins to harbor a potential cNLS (Anand et al., 2025). In the study, *in-silico* docking analysis of ribosomal protein L34 and TnpB with importin-α was performed. Such docking prediction was also proposed using NLScore (Hari et al., 2017). Transposases can be exploited for targeted gene transfer into mammalian cells (Kahlig et al., 2010). Among the putative *S. aureus, E. faecium, and H. pylori* NMs, we also identified IS200/IS605 family transposases (**Supplementary Table 1**), which did not proceed to the final table shown above. However, these RNA guided transposases/nucleases are the predecessors of the CRISPR-Cas12 family of DNA binding enzymes (Druteika et al., 2025; Karvelis et al., 2021; Wu et al., 2024). Accordingly, a functional SaCas9 has been identified for *S. aureus* (Ran et al., 2015), while this protein was not annotated in any of our analyzed isolates. Similarly, Cas9 was only annotated in *E. faecium* genome LFY64. This discrepancy highlights that coding and production of bacterial NMs may be highly isolate-specific.

We identified ribosomal protein L34 in *S. aureus, E. faecium*, *E. faecalis*, and *A. baumannii*. Similarly, in most species we used, transposases were identified (not in *S. aureus* and *H. pylori*), besides other DNA/RNA binding/unwinding proteins (e.g. helix turn-helix and DEAD-box containing) or nucleases. Assuming that these are released by bacteria and enter the nucleus, they may modulate host genetic and epigenetic processes. Of note, ribosomal proteins formed the largest group of putative NLS-containing proteins in all analyzed species. Similar proteins were also among the candidate list of proteins identified using a proximity-biotinylation approach upon infection of bovine macrophage-like cells with *M. bovis* (Lu et al., 2024). Interestingly, for many prokaryotic and eukaryotic ribosomal proteins ‘moonlighting’ functions have been described besides their role as part of the ribosome (Das et al., 2025; Ochkasova et al., 2023).

The second most common group identified comprised proteins involved in the regulation of bacterial cell division (e.g. FtsK, Ebh, DamX, or M23/M37 hydrolases). FtsK and DamX are part of the bacterial divisome (Soderstrom et al., 2022; Veiga et al., 2023), which also includes the bacterial tubulin homologue FtsZ (Paul and Voth, 2025). This may suggest a link to nuclear chromatin organization (Chumova et al., 2019; Kristensson, 2021). For membrane anchored M23/M37 hydrolases (Razew et al., 2022), or Ebh we currently have no obvious indication for intranuclear functions, unless they harbor hidden functions not yet recognized. Among the predicted is the GTPase-regulating protein YihI (**Table 5**). In the other organisms analyzed, various GTP-binding, GTPases or GTPase-regulating proteins were predicted as well (see **Supplementary Table 1**), although, not always by all available tools. Such enzymes are partially conserved across the kingdoms of life (Caldon and March, 2003; Verstraeten et al., 2011), and may be exploited to hijack eukaryotic processes ranging from epigenetic regulation to membrane trafficking and organization, including nuclear envelope formation (Gasparotto et al., 2022; Matchett et al., 2014; Yokoyama and Gruss, 2013). They therefore constitute an attractive set of putative target NMs in the context of antimicrobial resistance (Shanbhag and Saraogi, 2023).

For many NM candidate proteins identified in our screening, homologues exist in other bacterial species. NLS-prediction should therefore also be performed for these proteins to validate the findings and to identify possible co-evolutionary mechanisms.

One caveat when applying in-silico predictions is that hydrophobic regions may mask NLS motifs, which may lead to in false positives or false negatives (van Zee et al., 1991; Wong et al., 2010).

Besides the putative release from intracellular pathogens and thus direct secretion into host cells, NMs could also be cargo of bacteria-derived extracellular vesicles (bEV). For instance, *A. baumannii* derived Tpn was identified as bEV cargo and induced methylation in human A549 cells (Ran et al., 2015). *F. nucleatum* outer membrane protein FomA is another protein found in bEV, which triggers NFκB responses in human gut epithelial cells (Martin-Gallausiaux et al., 2020); however, in this case, the response is elicited by TLR2 binding and not by nuclear translocation.

We analyzed cNLS first, however, proteins without and encoded NLS may be transported into the nucleus through other mechanisms, for example by diffusing through nuclear pores, as hypothesized for Mtb Rv3423 (Jose et al., 2016) or *E. coli* NleG5-1 (Valleau et al., 2018), or upon post translational modification (Hanford et al., 2021; Korgaonkar et al., 2025). *S. flexneri* NM OspF has been shown to require SUMOylation to enable its nuclear entry (Jo et al., 2017). Various bacteria exploit and target SUMOylation and other PTM (e.g. phosphorylation, acetylation, methylation, or ubiquitination) to modulate the effector function of proteins, including, e.g. SpCas9 (Ashida and Sasakawa, 2017; Sahin, 2026; Srikanth and Verma, 2017). *O. tsutsugamushi* Ank domain-containing proteins have been shown to modulate host cell apoptosis by regulating nuclear levels of the ubiquitin ligase cullin-1 (Allen et al., 2025). Various other post-translational modified bacterial proteins have recently been discussed in comprehensive reviews (Popa et al., 2016; Ribet and Cossart, 2018).

The predicted nuclear localization of candidate nucleomodulins has to be verified in *in-vitro* experiments, monitoring the protein expression, subcellular localization, and possible impact on cell health and viability.

In summary, this in silico analysis identified several candidate bacterial proteins harboring canonical nuclear localization signals (cNLS) across diverse ESKAPE pathogens, suggesting a potential role as nucleomodulins that may hijack host nuclear processes during intracellular infection. While our predictive pipeline successfully identified robust candidates, the functional relevance of these effectors in host-pathogen interactions requires rigorous experimental validation in relevant cellular models. These findings provide the basis for future studies to elucidate how these bacterial proteins modulate host chromatin dynamics and transcriptional responses, thereby offering new insights into the intracellular survival strategies of clinically important multidrug-resistant pathogens.

## Data Availability

The original contributions presented in the study are included in the article/**Supplementary Material**. Further inquiries can be directed to the corresponding author.
